# Covalent Linkage of BODIPY‐Photosensitizers to Anderson‐Type Polyoxometalates Using CLICK Chemistry

**DOI:** 10.1002/chem.202102897

**Published:** 2021-09-29

**Authors:** Seda Cetindere, Simon T. Clausing, Montaha Anjass, Yusen Luo, Stephan Kupfer, Benjamin Dietzek, Carsten Streb

**Affiliations:** ^1^ Institute of Inorganic Chemistry I Ulm University Albert-Einstein-Allee 11 89081 Ulm Germany; ^2^ Department of Chemistry Faculty of Science Gebze Technical University 41400 Gebze/Kocaeli Turkey; ^3^ Helmholtz-Institute Ulm Helmholtzstr. 11 89081 Ulm Germany; ^4^ Institute of Physical Chemistry and Abbe Center of Photonics Friedrich-Schiller University Jena Helmholtzweg 4 07743 Jena Germany; ^5^ Department Functional Interfaces Leibniz Institute of Photonic Technology (IPHT) Albert-Einstein-Strasse 9 07745 Jena Germany; ^6^ Center for Energy and Environmental Chemistry Jena (CEEC-Jena) Friedrich-Schiller University Jena Philosophenweg 7a 07743 Jena Germany; ^7^ Current address: Department of Chemistry and Pharmacy Interdisciplinary Center for Molecular Materials (ICMM) Friedrich-Alexander-Universität Erlangen-Nürnberg Egerlandstr. 3 91058 Erlangen Germany; ^8^ Institute of Physical Chemistry Friedrich-Schiller University Jena Helmholtzweg 4 07743 Jena Germany

**Keywords:** BODIPY, organo-functionalization, polyoxometalates, photophysics, self-assembly

## Abstract

The covalent attachment of molecular photosensitizers (PS) to polyoxometalates (POMs) opens new pathways to PS‐POM dyads for light‐driven charge‐transfer and charge‐storage. Here, we report a synthetic route for the covalent linkage of BODIPY‐dyes to Anderson‐type polyoxomolybdates by using CLICK chemistry (i. e. copper‐catalyzed azide‐alkyne cycloaddition, CuAAC). Photophysical properties of the dyad were investigated by combined experimental and theoretical methods and highlight the role of both sub‐components for the charge‐separation properties. The study demonstrates how CLICK chemistry can be used for the versatile linkage of organic functional units to molecular metal oxide clusters.

## Graphical abstract

**Covalent functionalization o**f Anderson‐polyoxometalates with BODIPY light‐absorbers using CLICK chemistry is reported. The resulting photosensitizer‐polyoxometalate dyads are characterized by photophysical and theoretical methods, highlighting the electronic interactions between the sub‐components, and their possible application in visible light‐driven chemistry.

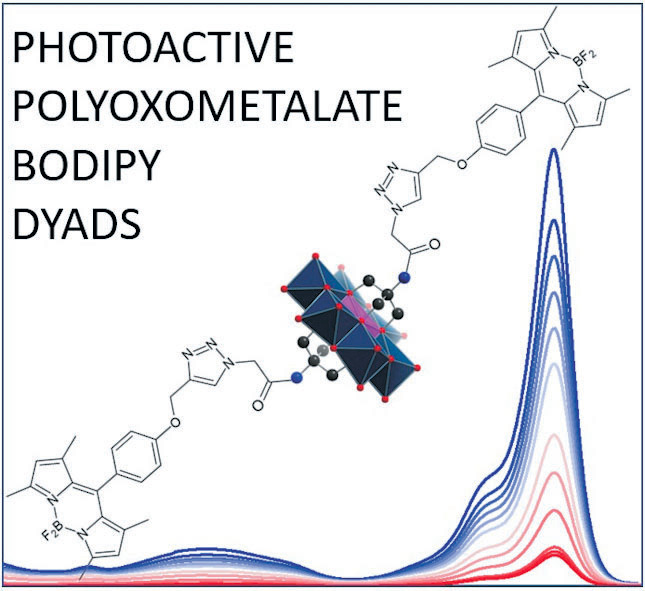

## Introduction

Covalently linked organic‐inorganic hybrid molecules are important multi‐functional materials for nanostructure design, catalysis, and sustainable energy.[^1^, ^2^] These systems can feature novel, synergistic properties which the individual components do not, thereby opening new avenues for materials design.^[3]^

In molecular materials chemistry, polyoxometalates (POMs) have emerged as ideal candidates for the design of organic‐inorganic hybrids. POMs are molecular metal oxide anions based on early transition metals such as Mo, V, and W.^[4]^ Over recent years, the covalent functionalization of POMs with organic or metal complex groups has led to tremendous progress in functional organic‐inorganic hybrid materials.[^5^, ^6^, ^7^, ^8^, ^9^] This has led to breakthroughs in bioinorganic hybrids,^[10]^ supramolecular nanostructures[^11^, ^12^, ^13^] and electrochemical surface functionalization.^[14]^

Recently, organo‐functionalized POMs have attracted immense interest in the fields of energy conversion and storage.[^15^, ^16^, ^17^] Organofunctionalized POMs have been utilized in lithium‐ion batteries^[18]^ as well as redox‐flow batteries.^[19]^ In solar energy conversion, POMs covalently linked to photoactive groups have been studied for charge‐separation and charge transfer/charge storage: In pioneering studies, Proust, Izzet and colleagues used Keggin‐^[20]^ and Dawson‐type^[21]^ polyoxotungstates as platforms for the covalent anchoring of metal complexes or organic photoactive groups,^[22]^ using stannyl^[20]^ or silyl^[21]^ linkages. The resulting systems showed visible light‐driven charge‐separation[^20^, ^22^] and hydrogen evolution.^[21]^ Building on these studies, Izzet, Gibson and co‐workers developed BODIPY (boron dipyrromethene)‐functionalized Keggin‐anions as photoactive species for rapid charge‐separation and long charge‐separated state stabilization. The authors propose that the systems are well suited for incorporation in photoelectrochemical devices.[^22^, ^23^] Related studies, pioneered by Harrimann, Ruhlmann, Lacote, Hasenknopf and others, have focused on the covalent linkage of organo‐functionalized POMs with metalated porphyrins,[^24^, ^25^] and have used these systems for the development of hybrid organic‐inorganic polymer surface coatings,^[26]^ as well as copolymer films for photocurrent generation.^[27]^ Following these seminal works, Streb and co‐workers have used Anderson‐molybdate POMs for the covalent anchoring of Ir‐photosensitizers. The team demonstrated that variation of the central heterometal (Fe^3+^, Co^3+^, Mn^3+^) of the Anderson anion can be used to tune the light‐driven hydrogen evolution of these systems. Further, detailed time‐resolved optical spectroscopy and spectro‐electrochemistry demonstrated the charge‐separation pathways^[28]^ and identified limiting processes for the hydrogen evolution catalysis.^[29]^

In particular, the use of noble‐metal‐free photosensitizers such as BODIPY and its derivatives[^30^, ^31^] has recently led to much progress in light‐driven charge‐separation and applications thereof. This is due to the versatile photophysical properties, high chemical stability, as well as easy and versatile chemical modification of BODIPY.[^30^, ^31^] Consequently, BODIPY derivatives have been employed in light‐driven hydrogen evolution,^[32]^ CO_2_ reduction,^[33]^ and water oxidation.^[34]^

Taking inspiration from these studies, we here report a CLICK‐chemistry based system which enables the covalent linkage of an azide‐functionalized Anderson type polyoxometalate **1** with two alkyne‐functionalized BODIPY **2** to give a covalent BODIPY‐Anderson‐dyad **3**. This concept is based on earlier studies by Oms, Dessapt, Mialane and co‐workers, who demonstrated that CLICK‐chemistry can be used to access these types of dyads.^[35]^ CLICK chemistry was chosen as it is selective and often quantitative, and offers a broad scope in terms of compounds and reaction conditions. In addition, CLICK reactions yield a stable 1,2,3‐triazole, which is stable under harsh reaction conditions.^[24]^

Here, we report the first example of a bis‐BODIPY‐Anderson‐POM hybrid, together with experimental and theoretical insights into the photophysical properties of the system.

## Results and Discussion

### Synthesis and characterization of the BODIPY‐POM‐dyad 3

**Figure 1:**
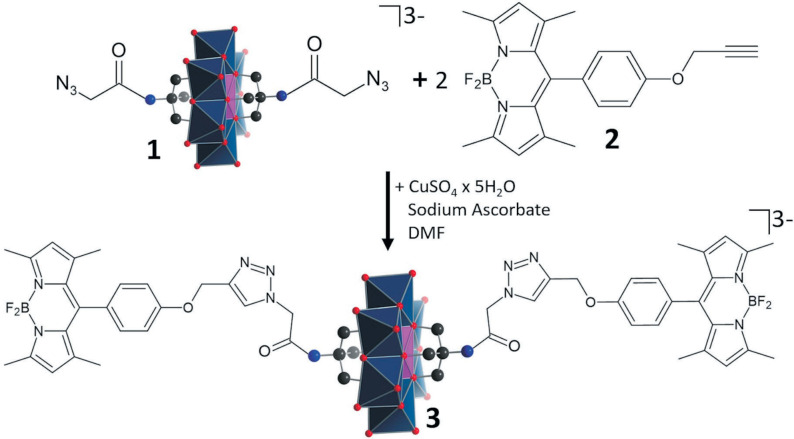
Schematic illustration of the CLICK‐chemistry based synthesis of the BODIPY‐POM dyad **3**.

**Figure 2:**
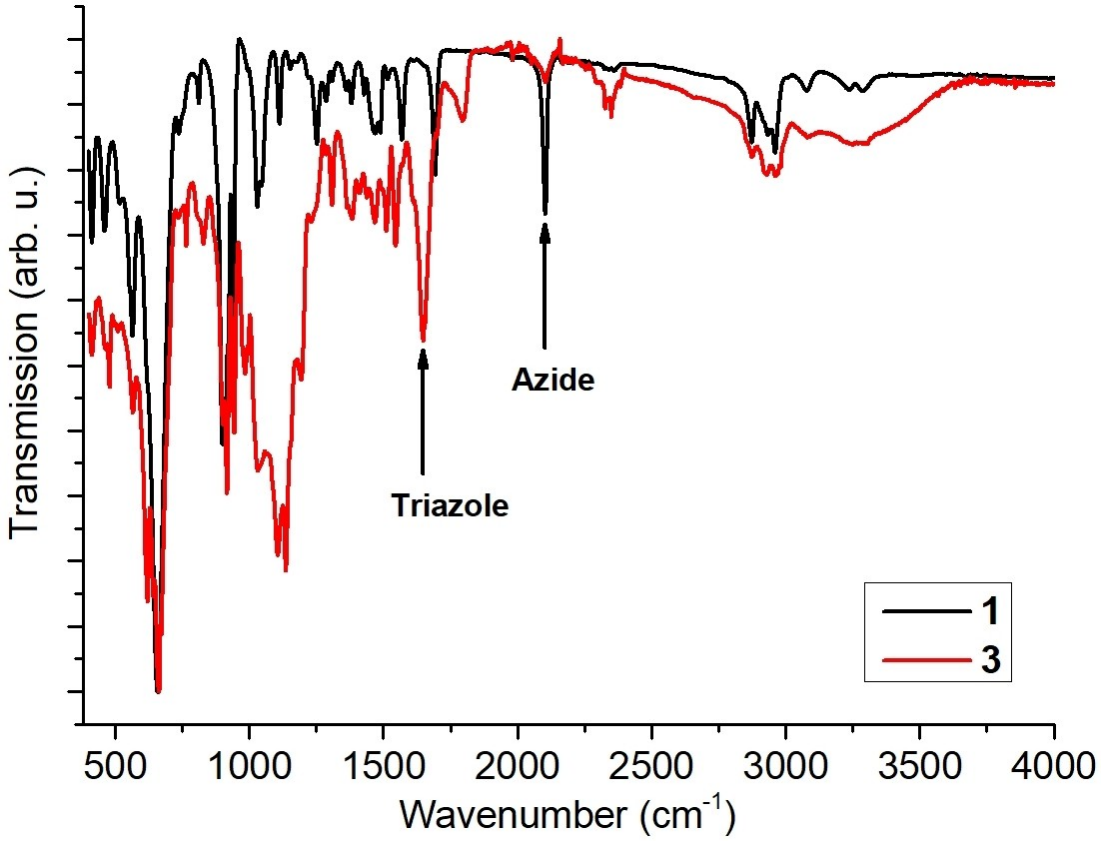
FTIR spectra of compounds **1** and **3**.

The BODIPY‐POM hybrid **3** was synthesized as follows: the literature‐known bis‐azide‐functionalized Anderson‐POM, (*n*Bu_4_N)_3_[MnMo_6_O_18_((OCH_2_)_3_C_3_H_3_N_4_O)_2_] **1**,^[36]^ was reacted with 4,4‐difluoro‐8‐(4’‐(prop‐2‐ynyloxy)phenyl)‐1,3,5,7‐tetramethyl‐4‐bora‐3a,4a‐diaza‐s‐indacene (BODIPY, **2**)^[37]^ in water‐free, de‐aerated dichloromethane using CuSO_4_ ⋅ 5H_2_O as CLICK catalyst and sodium ascorbate as reducing agent (Figure 1, synthetic details see Supporting Information). The bis‐BODIPY‐functionalized Anderson‐POM (*n*Bu_4_N)_3_[MnMo_6_O_28_C_56_H_60_B_2_F_4_N_12_] **3** was obtained in yields of 82 % (based on **1**). The identity and purity of **3** was confirmed by elemental analysis, ^1^H (Supporting Information, Figure S1) and ^19^F NMR spectroscopy (Supporting Information, Figure S2), electrospray ionization mass spectrometry (ESI MS, Supporting Information, Figure S3) and matrix‐assisted laser desorption/ionization mass spectrometry (MALDI MS, Supporting Information, Figure S4). No copper catalyst residues were observed in the solid samples of **3**, based on energy dispersive X‐Ray (EDX) analysis (Supporting Information, Figure S5). FTIR spectroscopy (Figure 2) showed that the characteristic azide stretching vibration observed for the POM‐precursor **1** (2100 cm^−1^) is absent in **3**, while a prominent triazole band at 1680 cm^−1^ is observed, indicating the successful formation of the triazole‐linked species.

### Electrochemical analyses

**Figure 3:**
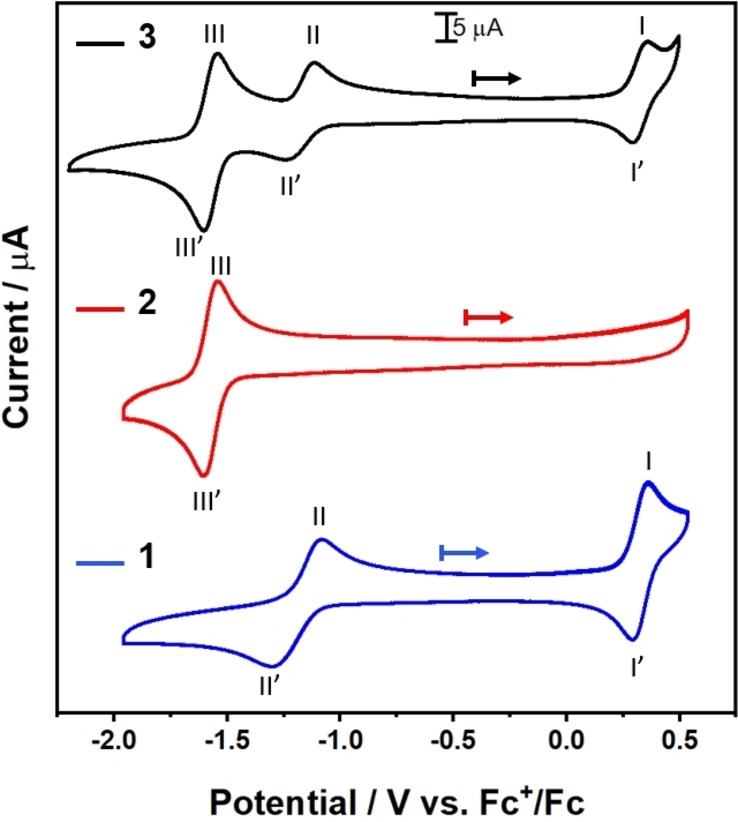
CV data for **1, 2** and **3**. Conditions: water‐free, de‐aerated DMF containing 0.1 M (*n*Bu_4_N)PF_6_, scan rate: 0.1 V s^−1^, [analyte]: 1 mM.

Cyclovoltammetric (CV) analysis of **1**, **2** and **3** was performed to assess accessible redox transitions in these species. As shown in Figure 3, the POM‐precursor **1** shows two redox transitions, assigned to the Mn^III/II^ couple (I/I’, *E*_m_=0.32 V, all potentials given vs. Fc^+^/Fc), and one Mo^VI/V^ couple (II/II’, *E*_m_=−1.20 V), respectively.^[38]^ The BODIPY precursor **2** shows one quasi‐reversible process (III/III’) at *E*_m_=−1.58 V. In the dyad **3**, the three processes are retained with virtually no potential changes, i. e. *E*_m_=0.33 V (I/I’), *E*_m_=−1.18 V (II/II’), and *E*_m_=−1.57 V (III/III’).

### Quantum chemical simulations

**Figure 4:**
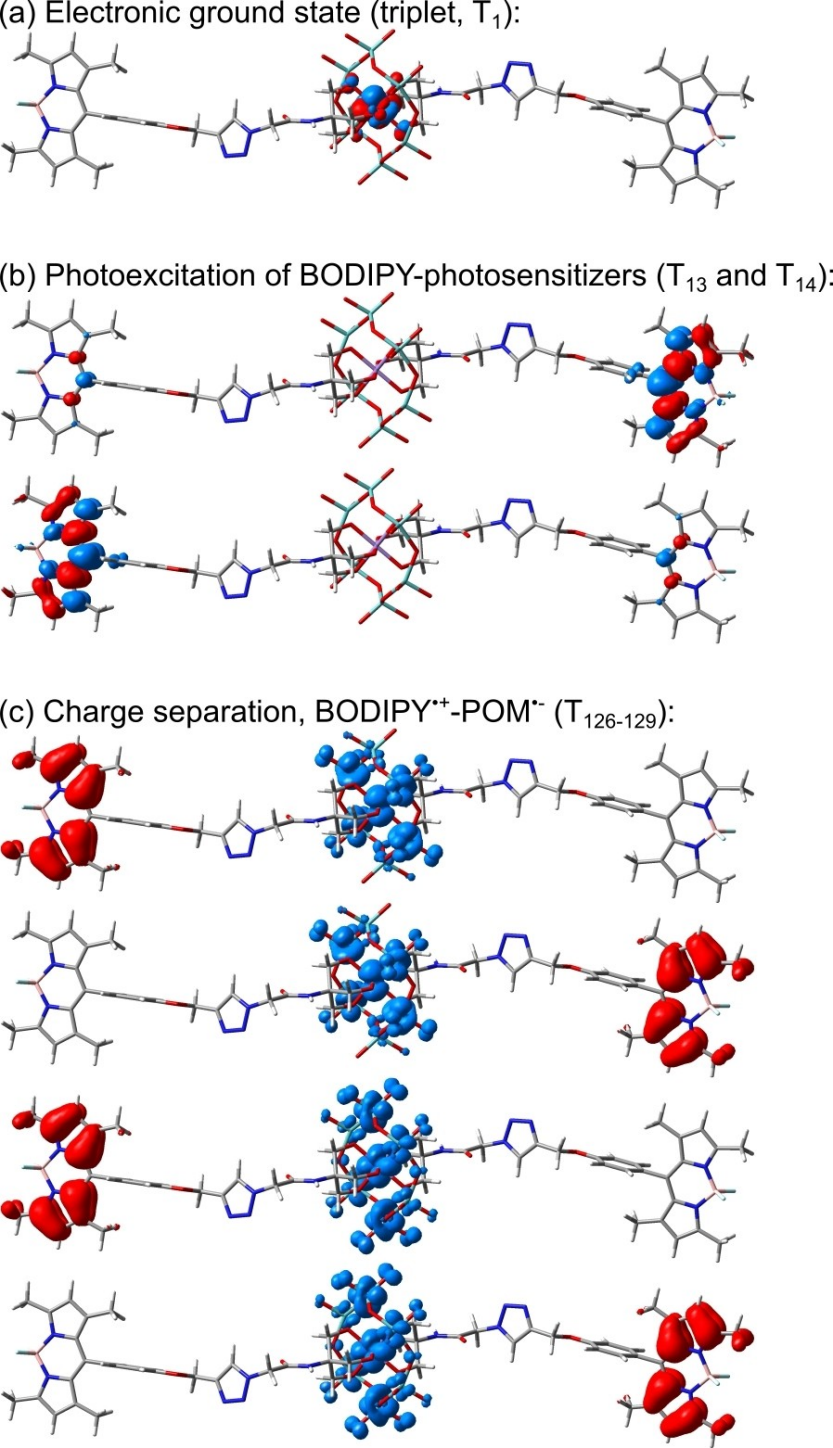
(a) Electronic ground state configuration of BODIPY‐POM dyad **3** with the central manganese(III) visualized by the spin density as obtained at the density functional level of theory (DFT; PBE0/def2‐SVP)[^39^, ^40^, ^41^] in acetonitrile. The triplet species of **3**, i. e. formal Mn^III^ configuration (3d_xy_)^2^, (3d_xz_)^1^, (3d_yz_)^1^, (3d_x2‐y2_)^0^, (3d_z2_)^0^, is favored by 1.76 eV compared with the closed‐shell singlet species, i. e. (3d_xy_)^2^, (3d_xz_)^2^, (3d_yz_)^0^, (3_dx2‐y2_)^0^, (3d_z2_)^0^. (b) Strong dipole‐allowed excitations (into T_13_ and T_14_) for the initial photoexcitation of BODIPY photosensitizers in **3** as predicted at the time‐dependent DFT (TDDFT) level of theory, illustrated by charge density differences (CDDs). (c) Charge‐separated states (T_126_‐T_129_) accessible upon photoinduced electron transfer from the excited BODIPY photosensitizer(s) (T_13_ and T_14_) to the POM. Charge transfer takes place from red to blue.

To evaluate the electronic structure of the BODIPY‐POM dyad **3** at the molecular level, quantum chemical simulations were performed at the density and time‐dependent density functional (DFT and TDDFT) levels of theory. Initially, the nature of the electronic ground state was evaluated, i. e. with respect to the configuration of the manganese(III) centre of the POM. In case of Mn^III^ with a formal 3d^4^ configuration two configurations are conceivable – either a closed shell singlet, or an (opened shell) triplet species. In the first case, the closed shell singlet species features a formal Mn^III^ electronic configuration of (3d_xy_)^2^, (3d_xz_)^2^, (3d_yz_)^0^, (3d_x2‐y2_)^0^, (3d_z2_)^0^ with one vacant t_2g_ orbital, for example the d_yz_ orbital. In the second case, the triplet species exhibits two unpaired electrons in the t_2g_ level, yielding the following configuration of the manganese(III): (3d_xy_)^2^, (3d_xz_)^1^, (3d_yz_)^1^, (3d_x2‐y2_)^0^, (3d_z2_)^0^, as illustrated by the spin density shown in Figure 4a. In case of **3**, DFT simulations performed at the PBE0/def2‐SVP[^39^, ^40^, ^41^] level of theory and considering solvent stabilization (acetonitrile) by a polarizable continuum model reveal that the triplet species is energetically favoured with respect to the closed shell species by almost 1.8 eV.

Subsequently, the excited state properties withing the Franck‐Condon region – as given by fully optimized triplet ground state structure – were calculated using TDDFT. Thereby, the same computational protocol was applied as for the preceding ground state calculations. Noteworthy, TDDFT lacks accuracy regarding the description of multiconfigurational systems and boron‐species in general. Previous computational studies showed that the PBE0 functional is capable of providing a satisfying computational description of the low‐lying excited states in BODIPY dyes.[^32^, ^42^, ^43^, ^44^, ^45^, ^46^] For the present BODIPY‐POM dyad, TD‐PBE0 allows to assign the lowest strongly dipole and spin‐allowed ππ* excitations of the two BODIPY chromophores, i. e. T_13_ and T_14_ (at 422 nm or ∼2.9 eV) in Figure 4b, to the sharp absorption feature measured at 501 nm. Still, the typical overestimation of the excitation energy as obtained by TDDFT with respect to multi‐configurational reference data is observed.[^32^, ^42^, ^44^, ^46^] Besides the light‐harvesting properties, given by the ππ* excitations of the organic dyes (T_13_ and T_14_), the energetic position of charge‐separated states associated to the photoreduction of the POM, in particular of the central manganese from Mn^III^ to Mn^II^, are of potential interest in the scope of photocatalytic applications. Based on the electronic ground configuration of Mn^III^, both semi‐occupied d_Mn_‐orbitals (d_xz_ and d_yz_) can act as electron acceptor sites – as evident from the low‐lying doubly degenerate metal‐centered excited states in **3** (Supporting Information, Table S3). Therefore, four quasi‐degenerate charge‐separated states of BODIPY^.+^‐POM^.−^ character (T_126_‐T_129_ in Figure 4c) are obtained involving both dyes and both semi‐occupied d_Mn_‐orbitals. Further information with respect to the TDDFT results as well as regarding the computational protocol are collected in the Supporting Information, Section 6.

### Photophysical properties of BODIPY‐POM 3

**Figure 5:**
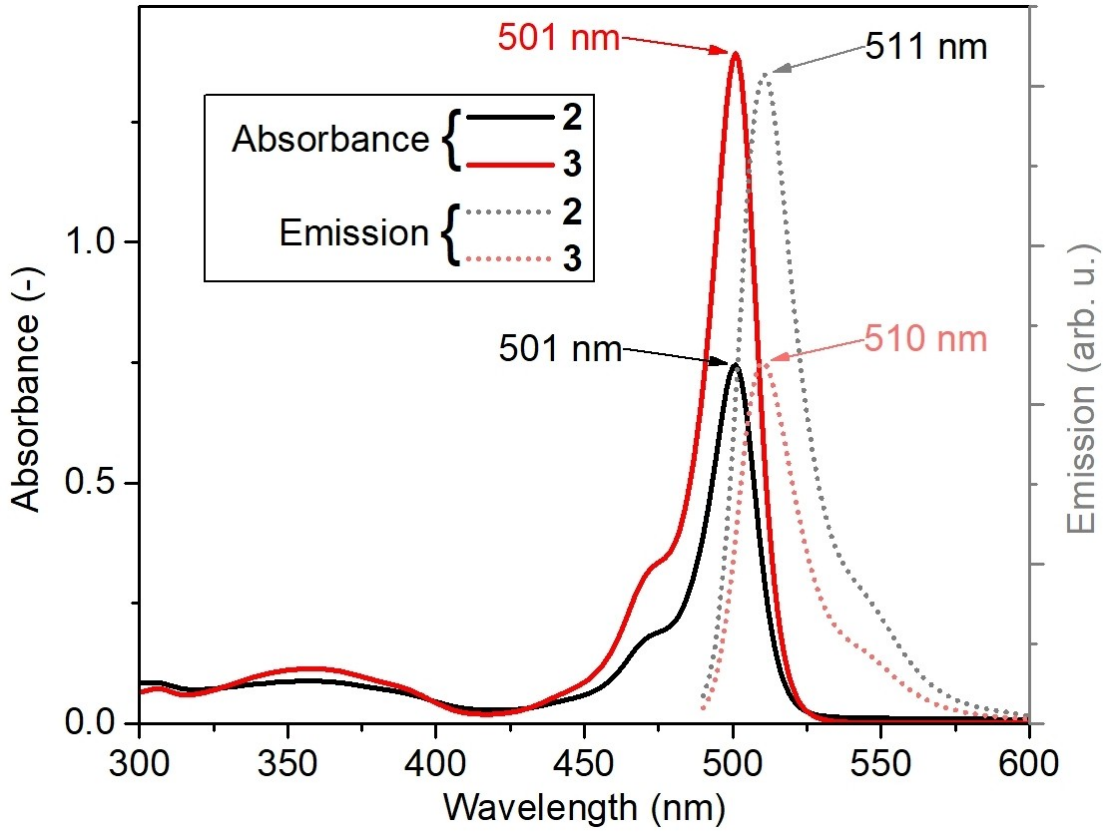
UV‐vis absorption and emission spectra of **2** and **3**. Concentrations: 10^−6^ M in water‐free, de‐aerated MeCN, λ_exc_=470 nm.

The photophysical properties of **3** were first investigated by steady‐state UV‐vis and emission spectroscopies and compared with the BODIPY precursor **2** (Figure 5). In the visible range, the absorption of **3** is dominated by an intense feature at ca. 500 nm, which is virtually identical to the absorption of the BODIPY precursor **2**. Quantum chemical simulations performed for **3** allow assigning this feature to two strongly dipole‐allowed ππ* excitations of the BODIPY dyes, see T_13_ and T_14_ in Figure 4b. In accordance, steady‐state emission spectroscopy of **3** shows a BODIPY‐based emission signal at λ_max_=510 nm with a shoulder at ca. 540 nm (Figure 5). An emission quenching of ca. 33 % is observed for the BODIPY‐POM **3** when comparing with the precursor **2**. Note that concentration‐dependent UV‐vis absorption and emission spectra of **2** (Supporting Information, Figures S6, S7) and **3** (Supporting Information, Figures S8, S9) in MeCN showed no changes of the absorption or emission wavelength maximum, indicating that under the experimental conditions used, no aggregation‐induced spectral changes are observed.

**Figure 6:**
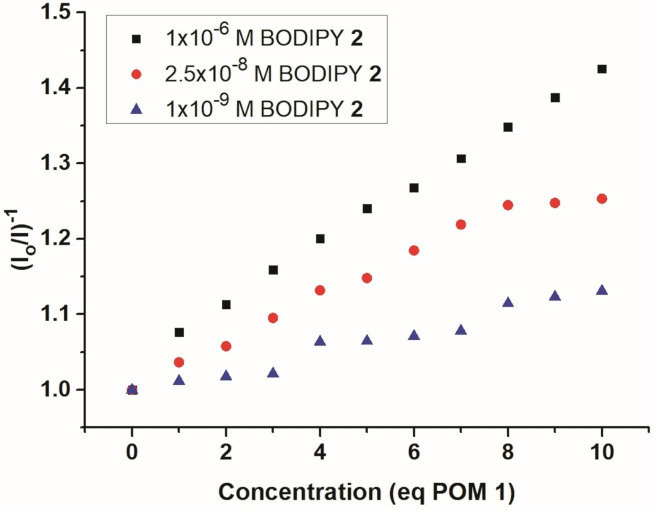
Stern‐Volmer plots showing concentration‐dependent emission quenching of the noncovalent system POM **1**/BODIPY **2**.

To gain insights into the photophysical differences between the non‐covalently linked system POM **1** / BODIPY **2** and the covalent hybrid **3**, emission quenching studies were performed at different concentrations of **2** (1 μM, 0.025 μM and 0.001 μM), and at different equivalents (1‐10 μM and 10–100 μM) of POM **1** (Supporting Information, Figures S10–S12). The corresponding Stern‐Volmer plots (Figure 6) show, that at low concentrations of **2**, virtually no emission quenching is observed. With increasing BODIPY concentration, emission quenching increases in a linear fashion, highlighting that energy/electron transfer between both species is highly concentration dependent.

### Time‐resolved spectroscopic characterization

Femtosecond transient absorption (TA) spectroscopy was performed to study the excited‐state properties of **2** and **3**. In the following, we will discuss the time‐resolved data using nomenclature that refers to isolated BODIPY photosensitizers, i. e. we refer to the optically active transitions as singlet transitions (on the BODIPY unit) despite the fact that calculations show that the entire system (including the POM) is in a triplet state due to unpaired electrons on the Mn ion. However, the optical properties of the dyads upon visible excitation can be discussed based on isolated BODIPY centers, i. e. photosensitizers which are electronically only very weakly coupled to the POM fragment of the dyads. Hence, for the sake of readability, the following section will adopt the nomenclature used in literature for isolated BODIPY fluorophores.

**Figure 7:**
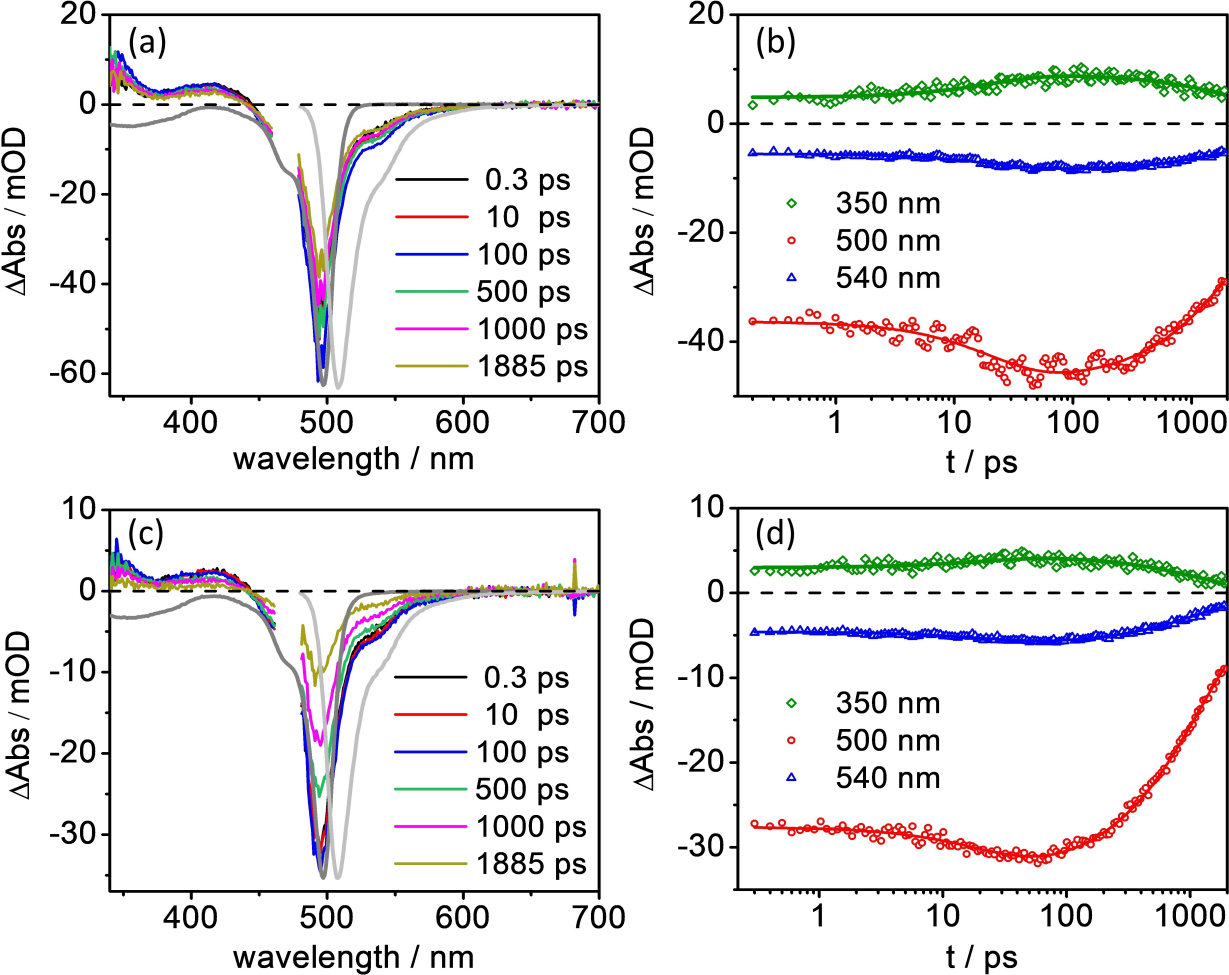
fs transient absorption spectra at selected delay times (left) and selected kinetic traces with the corresponding fits (right) obtained upon excitation at 475 nm in aerated acetonitrile for **2** (a, b) and **3** (c, d). The dark and light grey lines in (a) and (c) represent the inverted steady‐state absorption and emission spectra, respectively.

Upon excitation in the blue edge of BODIPY's ππ* absorption band at 475 nm, the fs TA spectra of **2** and **3** initially display identical spectral features (see Figure 7a, c). At 0.3 ps, the TA spectrum shows two excited‐state absorption (ESA) features between 340 and 445 nm as well as a strong negative peak stemming from (partially) overlapping ground‐state bleach (GSB) at around 500 nm and stimulated emission (SE). These spectral features are typically observed for BODIPY and related to the properties of the lowest energy ππ* (S_1_ – nomenclature referring to the isolated BODIPY) excited state.[^47^, ^48^, ^49^] Between 0.3 and 50 ps, the ESA (between 340 and 370 nm), GSB (at around 500 nm) and SE (at ca. 540 nm) increase in intensity (Figures 7b and d), likely associated with vibrational relaxation within the ππ* state. Between 100 ps and 2 ns, the spectral shape remains constant and an overall decay of the ππ* state is observed for **2** and **3** (Figures 7a and c). Compared to **2**, a faster decay of this BODIPY centered ππ* state is seen in **3** (Figures 7b and d). However, no new species, for example a charge‐separated state BODIPY^.+^‐POM^.−^, is spectrally captured in the time regime studied: the TA spectra of **2** and **3** recorded at the longest available delay time are identical (see Figure S13). As predicted by the performed TDDFT simulations, the four quasi‐degenerate charge‐separated states, i. e. T_126_, T_127_, T_128_ and T_129_ in Figure 4b, are found at high excitation energies of approximately 4.57 eV (271 nm) within the Franck‐Condon region.

For quantitative analysis of the fs TA results, decay‐associated spectra (DAS)[^50^, ^51^] were generated based on global multi‐exponential fits of the fs TA data. For **2** and **3**, two time‐constants were used to fit the fs TA data. The first component (τ_1_=21–23 ps, Supporting Information, Figure S14) indicates an increase of the signal amplitude of the ESA, GSB and SE, and is attributed to the vibrational cooling of the initially excited ππ* state. The magnitude of this time constant is in good agreement with the literature for the vibrational relaxation (10–20 ps) of the first excited ππ* (S_1_) state of BODIPY chromophores.[^48^, ^52^] The nanosecond process describes the decay of this ππ* state. Note that due to the limited time window (∼1.9 ns) of the setup, the nanosecond time constant is underestimated. For **3**, τ_2_=1193 ps (i. e. k=8.4×10^8^ s^−1^) shows a slightly faster decay of the ππ* state than the parent chromophore **2** (τ_2_=2120 ps, k=4.7×10^8^ s^−1^). This is consistent with the reduced fluorescence quantum yield of **3** (QY=0.20) compared to **2** (QY=0.48).

**Figure 8:**
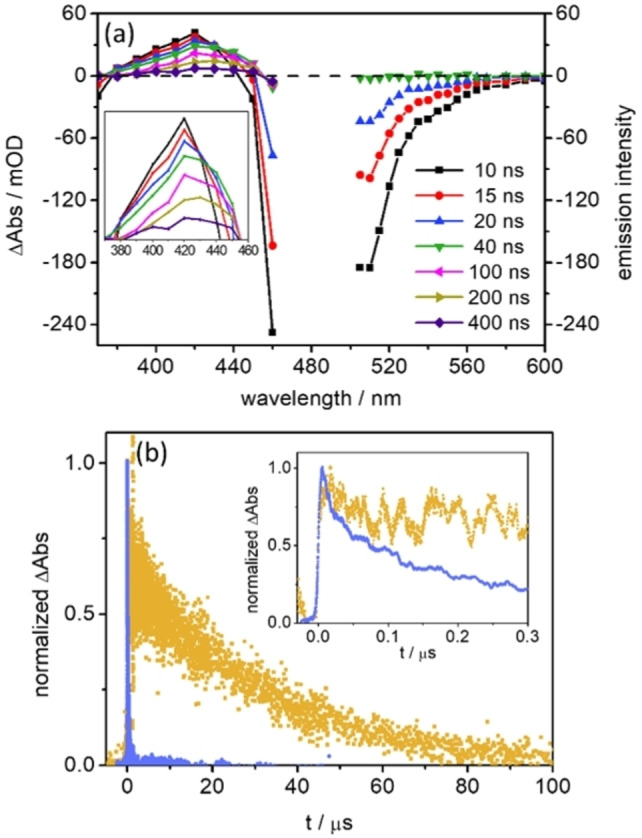
(a) ns transient absorption spectra (370–460 nm) and inverted time‐resolved emission spectra (505–600 nm) at selected delay times upon excitation of **3** at 475 nm in aerated acetonitrile. Inset: highlight of the excited‐state absorption between 370 and 460 nm. (b) Comparison of the normalized kinetics at 420 nm in aerated (light blue) and de‐aerated (light yellow) acetonitrile. Inset: enlargement of the time region to 0.3 μs.

Since the decay of the fs TA signal in **2** and **3** is not complete within the experimentally accessible range of delay times, nanosecond transient absorption spectroscopy was performed. Due to the very strong fluorescence of **2** and **3** between 500 and 700 nm, the ns transient absorption spectra were only recorded between 370 and 460 nm. In order to show the contribution from the fluorescence emission, time‐resolved emission spectra (between 500 and 600 nm) were recorded as well. Figure 8a shows the merged spectra of the transient absorption (370–460 nm) and the inverted time‐resolved emission (505–600 nm) of **3**. From 10 to 40 ns, the ESA intensity at 420 nm decreases and undergoes a fast red shift to ca. 430 nm (inset in Figure 8a). This spectral shift points to the conversion from the BODIPY ^1^ππ* state to the T_1_ state (please recall that this nomenclature relates to the electronic structure of the BODIPY fragment only).[^47^, ^53^] Thus, the emission signal from the BODIPY‐centred ππ* state decays completely within 40 ns (Figure 8a). Afterwards, the BODIPY T_1_ state decays to the ground state with a time constant of 240 ns in aerated acetonitrile (Supporting Information, Figure S15c). Very similar spectral evolution and kinetics were observed for **2** (Supporting Information, Figure S15a, b).

The two distinct processes, S_1_ → T_1_ and T_1_ → S_0_, are reflected in the kinetics of the transient absorption signal at 420 nm (inset in Figure 2b). A bi‐exponential decay is revealed in aerated acetonitrile (τ_ns1_<10 ns and τ_ns2_=240 ns). The generation of a triplet state in **3** was further confirmed by the significantly prolonged lifetime in de‐aerated acetonitrile: τ_ns2_=30 μs (Figure 8b and Figure S15d).

Thus, at a longer time regime, the presence of POM in **3** does not lead to a charge‐separated state (i. e. BODIPY^.+^‐POM^.−^) being detected, in agreement with the theoretical simulations which predict the charge‐separated states above 4.5 eV. Instead, a BODIPY localized T_1_ state is observed (Figure 8). The quantum chemical simulations performed exclusively within the Franck‐Condon point do not allow to assess the driving forces, reorganization energies and potential couplings associated to the photoinduced electron transfer among the donor and acceptor states of interest.^[54]^ Nevertheless, according to electrochemical data, oxidation of the BODIPY unit was not observed within the accessible potential window up to 0.5 V (vs. Fc^+^/Fc), suggesting a high energetic level (>1.68 eV) of the BODIPY^.+^‐POM^.−^ state. Thus, we conclude that upon excitation of **3**, BODIPY‐to‐POM electron transfer does not occur in a pure acetonitrile solution. Instead, a slightly faster intersystem crossing may take place, which (slightly) shortens the lifetime of the BODIPY S_1_ state. This is possibly due to the POM unit present in **3**.

## Conclusion

A novel Anderson‐polyoxomolybdate dyad covalently functionalized with two BODIPY groups is reported. Covalent attachment is possible by using a straight‐forward CLICK chemistry approach. Photophysical and electrochemical properties of the novel compound were investigated. According to these investigations, the dyad retains the unique photophysical properties of BODIPY, showing its promise as a photosensitizer in solar energy conversion processes. Future studies will include variation of the central metal to access a library of compounds based on this structural motif, which will allow modification of redox potentials for improved light‐induced electron transfer. Furthermore, modifications on the BODIPY moiety (e. g. functionalization with heavy elements for improved singlet‐triplet transition) will be performed to fine‐tune spectroscopic and photo‐/electrochemical properties, and to enable stable anchoring, for example on photoelectrode surfaces.

## Experimental Section

Synthetic, analytical, spectroscopic, and computational details are given in the Supporting Information.

## Conflict of interest

The authors declare no conflict of interest.

## Supporting information

As a service to our authors and readers, this journal provides supporting information supplied by the authors. Such materials are peer reviewed and may be re‐organized for online delivery, but are not copy‐edited or typeset. Technical support issues arising from supporting information (other than missing files) should be addressed to the authors.

Supporting Information
