# Supplementary material for: Covalent Linkage of BODIPY‐Photosensitizers to Anderson‐Type Polyoxometalates Using CLICK Chemistry
Source: Chemistry. 2021 Sep 29;27(68):17181–7. doi: 10.1002/chem.202102897 (PMC9292257; doi:10.1002/chem.202102897)
Supplement: Supplementary file 1 — Supporting Information [file CHEM-27-17181-s001.pdf]

Chemistry–A European Journal

Supporting Information

Covalent Linkage of BODIPY-Photosensitizers to Anderson-Type Polyoxometalates Using CLICK Chemistry

Seda Cetindere, Simon T. Clausing, Montaha Anjass, Yusen Luo, Stephan Kupfer,* Benjamin Dietzek,* and Carsten Streb*

1. Instrumentation

UV-Vis and emission spectroscopy: UV-vis spectroscopy was performed on a Varian Cary 50 spectrophotometer or JASCO V-670 spectrophotometer. All systems were measured in standard cuvettes (d = 10.0 mm). Emission spectra were recorded on a JASCO spectrofluorometer FP-8500 at room temperature.

^{1}H NMR spectroscopy was performed on a JEOL ECX 400 instrument at a sample temperature of 23°C. Chemical shifts, δ, are reported relative to residual ^{1}H resonances of the solvent in ppm, followed by peak multiplicity (s: singlet, d: doublet, m: multiplet), coupling constant *J* in Hertz, integration value and proton assignment.

^{19}F NMR spectroscopy was performed on a JEOL ECX 400 instrument at a sample temperature of 23°C. Chemical shifts, δ are reported in ppm.

FT-IR spectroscopy: FT-IR spectroscopy was performed on a Shimadzu IRPrestige-21 FT-IR spectrophotometer including a Golden Gate ATR unit. Signals are given as wavenumbers in cm^{-1} using the following abbreviations: vs = very strong, s = strong, m = medium, w = weak and b = broad.

Elemental analysis: CHN analysis was performed on a Elementar vario MICRO cube.

Electrospray ionization mass spectrometry (ESI-MS): MS measurements were performed on a Bruker solariX Hybrid 7T FT-ICR in ESI mode.

Matrix-assisted laser desorption/ionization mass spectrometry (MALDI MS): MALDI-MS measurements were performed on a Bruker solariX Hybrid 7T FT-ICR in MALDI mode.

Energy-dispersive X-ray spectroscopy (EDX): EDX measurements were performed on an SDD Apollo XV EDX detector (EDAX AMETEK).

Electrochemistry: DC cyclic voltammetry (CV) experiments were performed on a Pine Research WaveDriver 200 electrochemical workstation equipped with a standard three-electrode setup: working electrode: glassy carbon electrode (d = 3.0 mm), quasi reference electrode: Ag wire (in a glass frit containing electrolyte solution), counter electrode: Pt wire. All potentials are given relative to the ferrocene/ferrocenium internal standard. All experiments were performed in water-free, de-aerated DMF using $n\mathrm{Bu_4NPF_6}$ (0.1 M) as supporting electrolyte. The solutions were purged with argon for at least 15 min to remove O_2 and kept under a slight positive Ar pressure while performing the experiments."

Quantum yield determination. The absolute quantum yields of compounds **2** and **3** were recorded in acetonitrile using an integrating sphere. The absorbance was adjusted to Abs = 0.1 at the excitation wavelength (475 nm). The fluorescence spectra were recorded using a FLS980 spectrofluorimeter (Edinburgh Instruments).

fs transient absorption (TA) spectroscopy. fs TA spectra were collected using a previously reported custom-built pump-probe laser system which is based on an amplified Ti: Sapphire oscillator (1 kHz, 800 nm). Samples were excited by pump pulse centered at

475 nm (TOPAS-C, Lightconversion Ltd.). The power of the pump beam at the sample position was kept at 0.40 mW with a beam diameter of 720 μm, corresponding to 4.69 × 10^{18} photos m^{-2} per pump pulse. A white light supercontinuum generated by focusing a fraction of the fundamental in a CaF_2 plate is used to probe the samples in a spectral range between 340 and 700 nm. The pump beam is delayed in time with respect to the probe beam by means of an optical delay line and the polarization between probe and pump is set at the magic angle (54.7°). Fresh solution was used for the fs TA measurement with an optical density of 0.2 at 475 nm. The fs TA spectra were displayed after chirp correction. The fs TA data were analyzed by a global multi-exponential fit after exclusion of a temporal window of 200 fs around time-zero in order to avoid contributions of the coherent-artifact region to the data analysis. Furthermore, a spectral band of ca. 20 nm around the pump-wavelength is omitted from the data analysis due to pump-scatter in this spectral range. For all the time-resolved experiments, the stability of samples was ensured by recording the UV/Vis absorption spectra (JASCO V-670 spectrometer) at room temperature before and after each fs TA measurement.

ns transient absorption (TA) spectroscopy. ns TA spectra were collected applying pump pulses centered at 475 nm. The excitation pulses are produced by a Continuum OPO Plus which is pumped by an continuum surelite Nd:YAG laser system (pulse duration 5 ns, repetition rate 10 Hz). The probe light is provided by a 75 W xenon arc lamp. Spherical concave mirrors are used to focus the probe beam into the samples and then send the beam to the monochromator (Acton, Princeton Instruments) and detected by a photomultiplier tube (Hamamatsu R928). The signal is amplified and processed by a commercially available detection system (Pascher Instruments AB). For the ns TA measurements, the power of the pump pulses was kept at 0.3 mJ. Each sample was freshly prepared, and the optical density was set to ca. 0.3 at the excitation wavelength 475 nm. Due to the very strong fluorescence emission from the BODIPY, diluted solutions (optical density at 475 nm was adjusted to 0.08) and a significantly reduced power of the pump (0.3 μJ) was used for the time-resolved emission measurement. All measurements were performed in 1 cm path length fluorescence cuvettes. Oxygen-free solutions were obtained by five freeze-pump-thaw cycles.

General remarks: All chemicals were purchased from Sigma Aldrich, ABCR or ACROS and were of reagent grade. The chemicals were used without further purification unless stated otherwise. The azide-functionalized POM **1**[1] and the alkyne functionalized BODIPY **2**[2] were prepared according to published procedures.

2. Synthetic section:

2.1. Synthesis of BODIPY functionalized POM, $(n\mathrm{Bu_4N})_3[\mathrm{MnMo_6O_{28}C_{56}H_{60}B_2F_4N_{12}}]$ 3: Azido-functionalized POM **1** (80 mg, 1eq.) and alkyne-functionalized BODIPY **2** (60 mg, 4 eq.) were dissolved in dry DMF (10 mL). While stirring, $CuSO_4•5H_2O$ (5 mg, 0.5 eq.) and sodium ascorbate (15 mg, 2 eq.) were added to the solution. The reaction mixture was kept under argon atmosphere. The progress of the reaction was monitored by taking a sample from the reaction mixture and recovering the POM by precipitation in diethyl ether,

after which an IR absorbance spectrum was taken. The disappearance of the absorption peak at 2105 cm^{-1} assigned to the azido function was a clear indication for the progress of the cycloaddition reaction. When the reaction was complete, EDTA functionalized silica was added, and the reaction was stirred overnight or until all precipitate disappeared (if any appeared). The residue was filtered off and the clear solution was evaporated to dryness. A minimal amount of acetonitrile was added to dissolve the POM and the solution was added dropwise to an excess of diethyl ether. The precipitate was collected by centrifugation and subsequently the remaining solid was washed with water and diethyl ether and dried at air for several days. BODIPY-POM-BODIPY **3** was isolated as an orange solid (yield 82%, based on **1**).

Elemental analysis in wt. -% for $(C_{16}H_{36}N)_3$-$MnMo_6O_{28}C_{56}H_{60}B_2F_4N_{12}$ (calcd.): C 44.36 (44.51), H 6.51 (6.04), N 7.85 (7.47).

Characteristic IR bands (in cm^{-1}): 3287 (w), 3068 (w), 2961 (ν C-H, m), 2931 (ν C-H, m), 2871 (ν C-H, m), 1672 (ν C=O, s), 1538 (m), 1473 (m), 1405 (w), 1379 (w), 1317 (m), 1237 (w), 1190 (s), 1110 (m), 1050 (m), 979 (m), 940 (ν Mo=O, vs), 915 (ν Mo=O, vs), 899 (ν Mo=O, vs), 836 (m), 654 (ν Mo-O-Mo, vs, br), 559 (w), 532 (w).

^{1}H NMR (400 MHz, MeCN-$_{d3}$): δ (ppm) = 65.94 (s, br, 12H, -CH_2-O), 8.02 (s, 2H, triazolyl-H), 7.95 (d, 4H, H_{aro}), 7.25 (d, 4H, H_{aro}), 7.20 (br, 8H, H_{aro}), 6.11 (br, 2H, NH), 5.39 (d, 4H, OCH_2-), 3.11 (m, 19.2H, -NCH_2- (TBA)), 2.91 (br, 4H, -CH_2CO-), 2.79 (br, 4H, -CH_2-), 2.49 (s, 12H, CH_3), 2.26 (q, 8H, -CH_2-CH_3), 1.61 (m, 19.2H, -NCH_2CH_2- (TBA)), 1.46 (m, 31.2H, 19.2H, -$NCH_2CH_2CH_2$- (TBA) + 12H –CH_3), 0.99 (m, 40.8H, 28.8H -CH_3 (TBA) + 12H –CH_2-CH_3).

^{19}F NMR (400 MHz, MeCN-$_{d3}$): δ (ppm) = -145.5 (m).

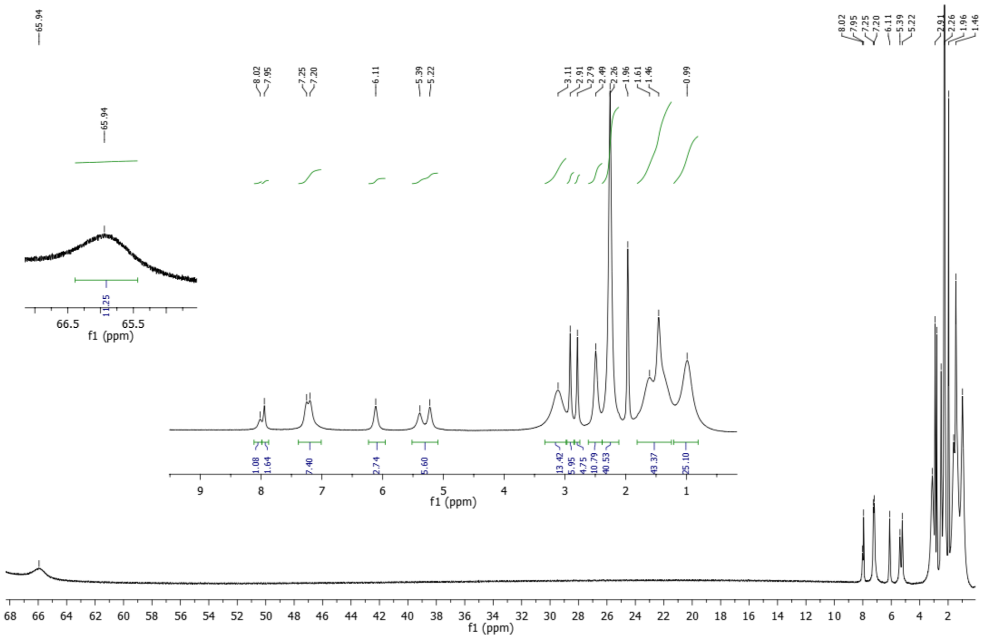

Figure S1. ^{1}H NMR spectrum of compound **3** (in acetonitrile-D_3).

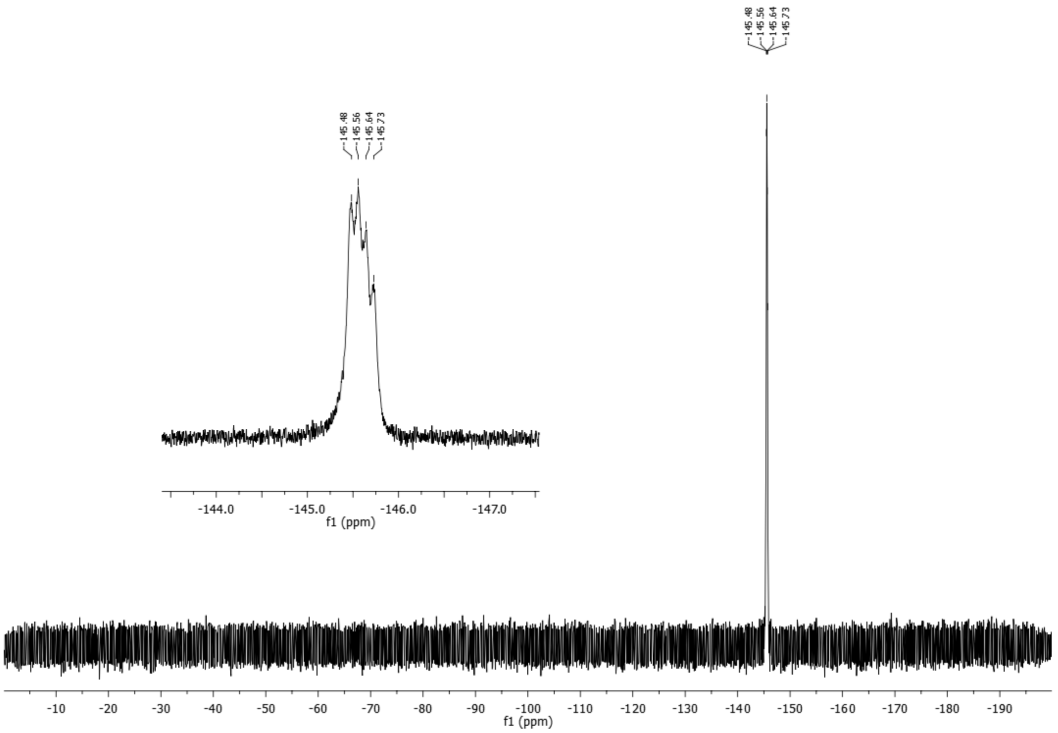

Figure S2. ^{19}F NMR spectrum of compound **3**.

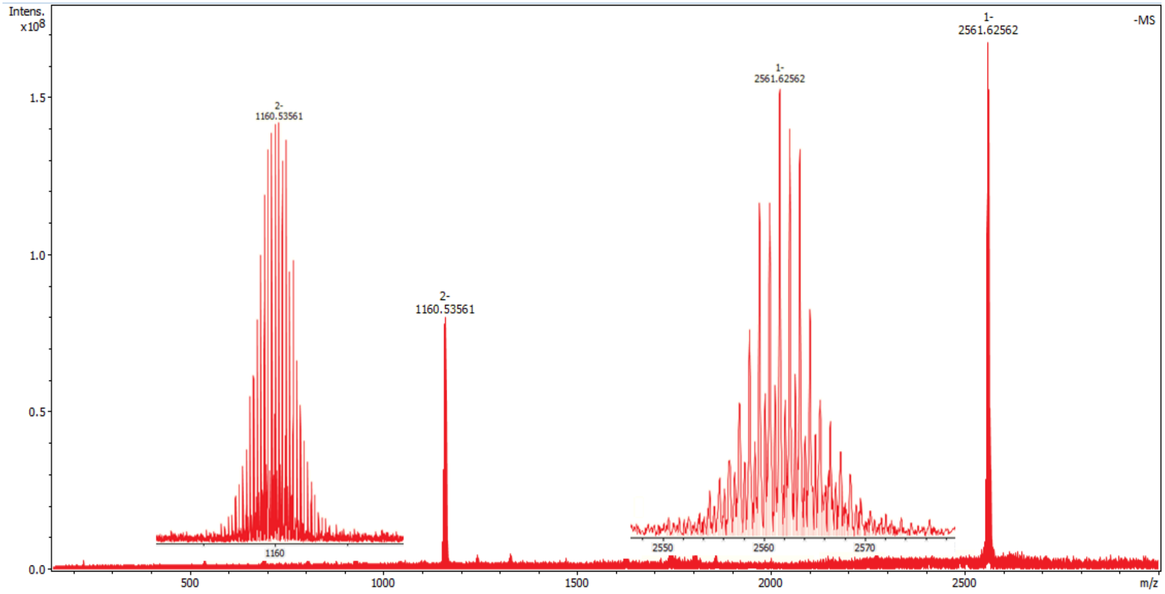

Figure S3. Negative-ion mode high resolution ESI mass spectrum of compound **3**, $(C_{16}H_{36}N)_3[MnMo_6O_{28}C_{56}H_{60}B_2F_4N_{12}]$.

Table S1: Peak assignments for the ESI mass spectrum of compound **3**.

Composition	Formula	Calculated (m/z)	Observed (m/z)
$[M\text{-}TBA]^{-}$	$C_{88}H_{132}B_2F_4MnMo_6N_{14}O_{28}$	2561.50	2561.63
$[M\text{-}2TBA+H]^{-2}$	$C_{72}H_{97}B_2F_4MnMo_6N_{13}O_{28}$	1160.53	1160.53

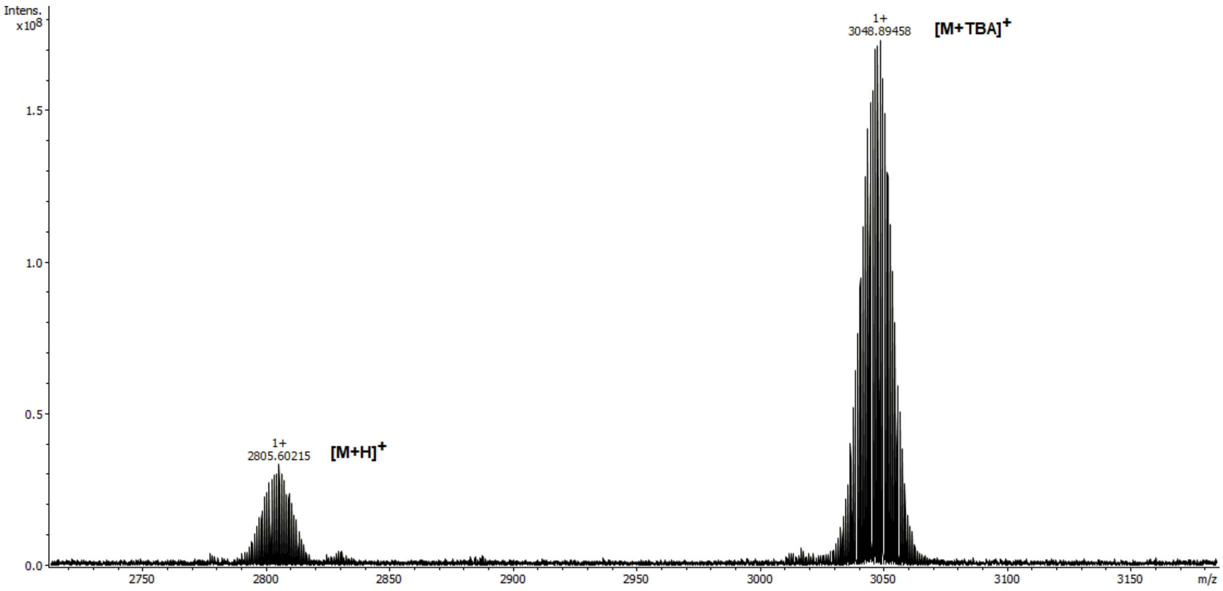

Figure S4. MALDI-MS spectrum of compound **3**, $(C_{16}H_{36}N)_3[MnMo_6O_{28}C_{56}H_{60}B_2F_4N_{12}]$.

Table S2: Peak assignments for the MALDI-MS spectrum of compound **3**.

Composition	Formula	Calculated (m/z)	Observed (m/z)
$[M+H]^{+}$	$C_{104}H_{168}B_2F_4MnMo_6N_{15}O_{28}$	2805.61	2805.60
$[M+TBA]^{+}$	$C_{120}H_{204}B_2F_4MnMo_6N_{16}O_{28}$	3048.88	3048.89

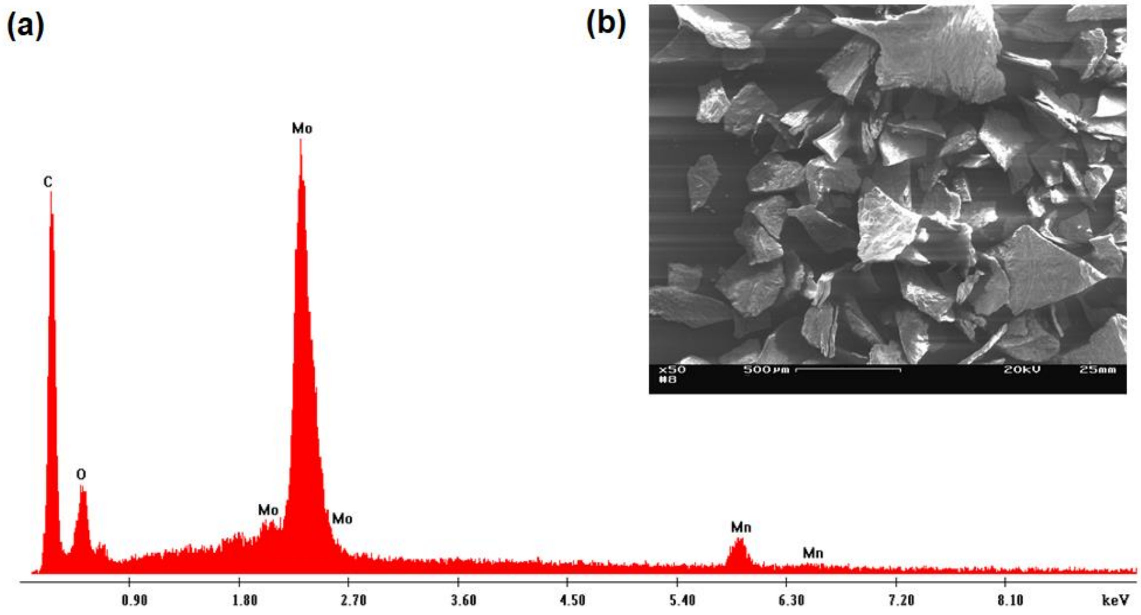

Figure S5. (a) EDX spectra and **(b)** SEM image of **3**

3. Photophysical Measurements

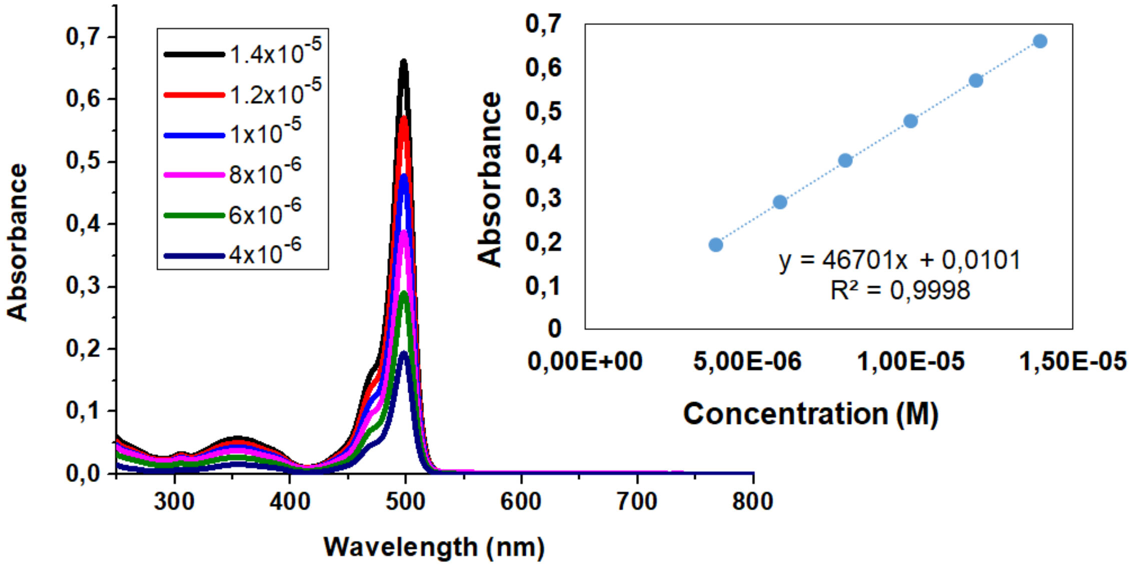

Figure S6. UV-Vis spectra of BODIPY **2** at different concentrations in MeCN.

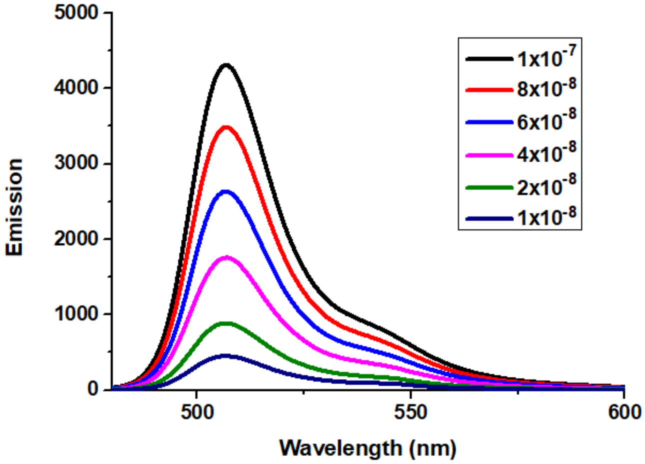

Figure S7. Emission spectra of BODIPY **2** at different concentrations in MeCN.

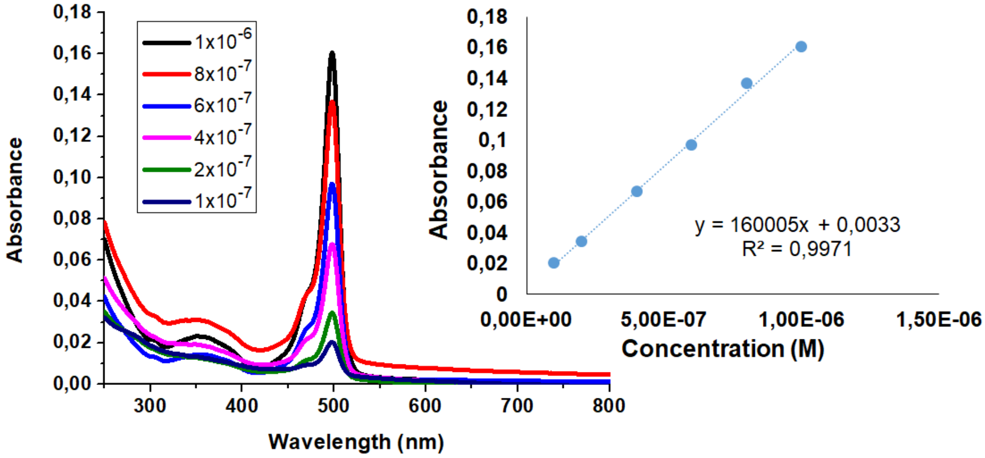

Figure S8. UV-Vis spectra of compound **3** at different concentrations in MeCN.

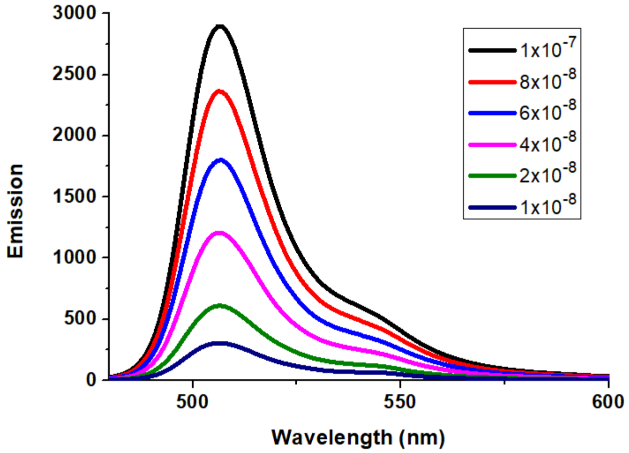

Figure S9. Emission spectra of compound **3** at different concentrations in MeCN.

4. Emission quenching measurements

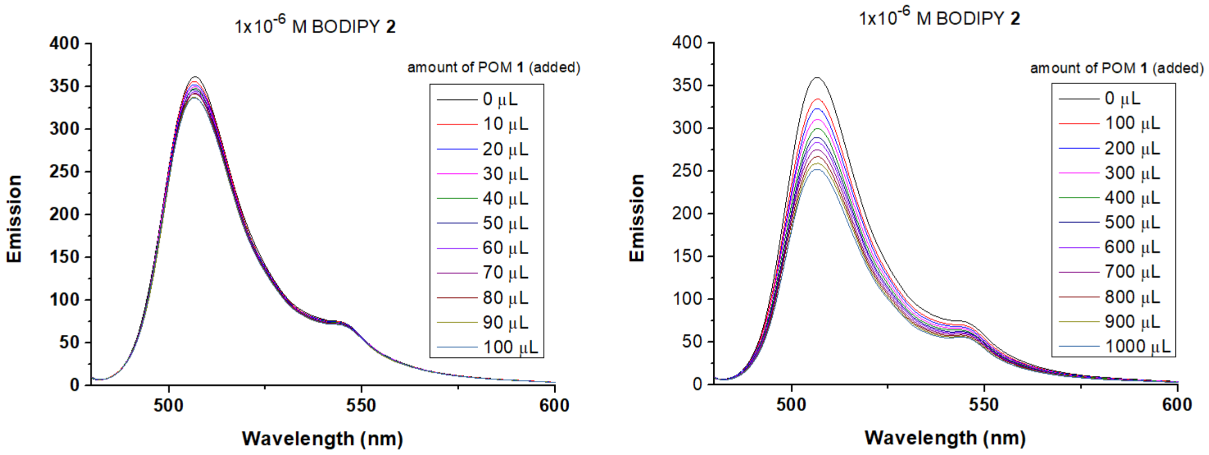

Figure S10. Emission quenching measurements for BODIPY **2** ($1x10^{-6}$ M) and POM precursor **1** (at different molar equivalents) in MeCN.

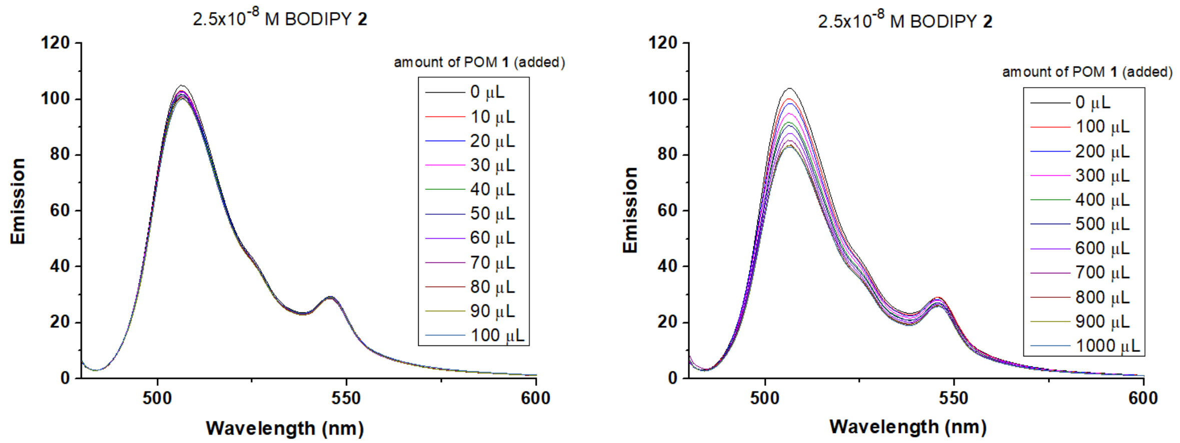

Figure S11. Emission quenching measurements for BODIPY **2** ($2.5x10^{-6}$ M) and POM precursor **1** (at different molar equivalents) in MeCN.

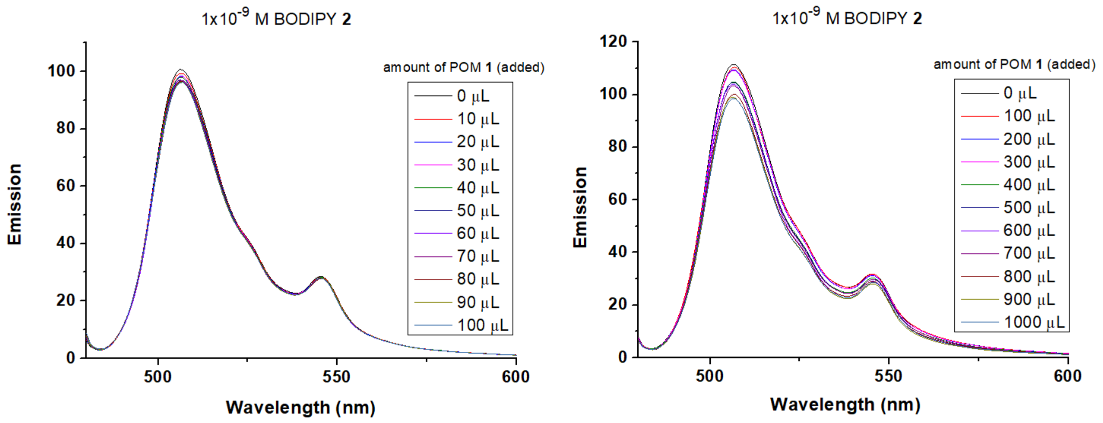

Figure S12. Emission quenching measurements for BODIPY **2** ($1x10^{-9}$ M) and POM precursor **1** (at different molar equivalents) in MeCN.

5. Time resolved spectroscopy

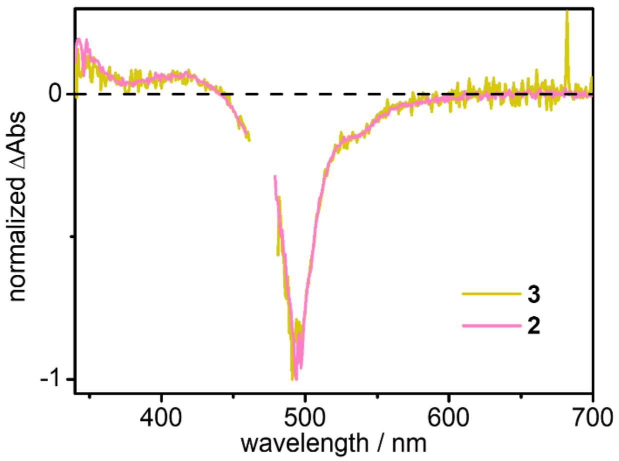

Figure S13. Comparison of the last fs transient absorption spectrum (delay time: 1885 ps) for **2** and **3**, $\lambda_{excitation}$ = 475 nm in aerated acetonitrile. The spectra were normalized at *ca*. 495 nm.

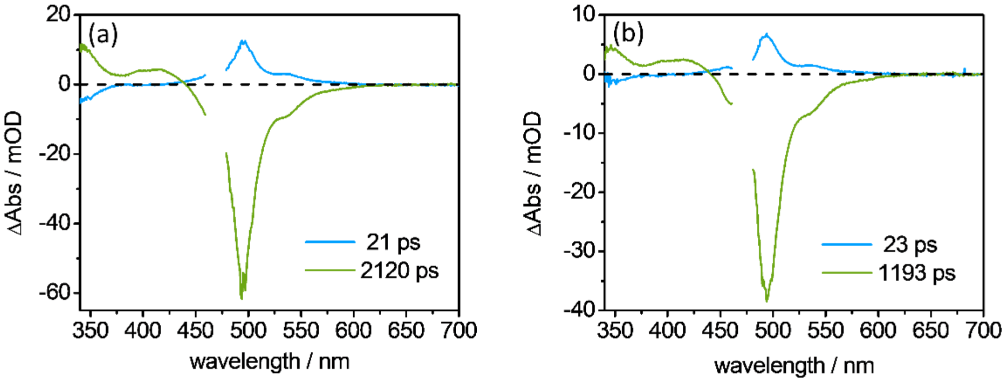

Figure S14. Decay-associated spectra for (a) **2**, and (b) **3**, based on the global fit of the fs TA data obtained upon excitation at 475 nm in aerated acetonitrile.

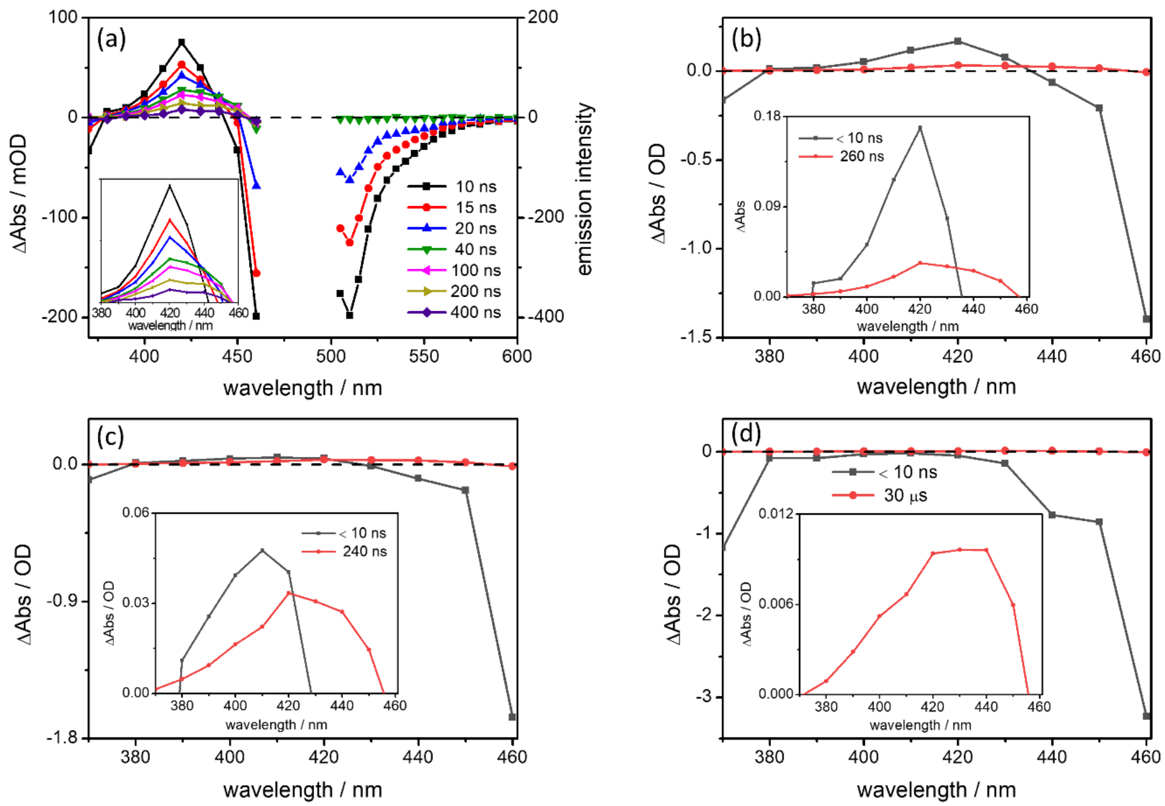

Figure S15. (a) ns transient absorption spectra (370-460 nm) and inverted time-resolved emission spectra (505-600 nm) at selected delay times upon excitation of **2** at 475 nm in aerated acetonitrile. Inset: highlight of the excited state absorption between 370 and 460 nm. (b) Global fitting results of the ns TA data (between 370-460 nm) of **2**. (c) and (d) Global fitting results of the ns TA data (between 370-460 nm) of **3** in aerated and de-aerated acetonitrile, respectively. Inset in (b-d): enlargement of the excited-state absorption bands.

6. Quantum chemical simulations

All quantum chemical calculations determining structural and electronic properties of the BODIPY-POM triad **3** were performed using the Gaussian 16 program.[3] Fully relaxed equilibrium geometries were obtained considering a singlet as well as a triplet ground state. Both electronic structures relate to a Mn^{III} species of $3d^4$ nature, while the triplet species, *i.e.* formally $(3d_{xy})^2$, $(3d_{xz})^1$, $(3d_{yz})^1$, $(3d_{x^2-y^2})^0$, $(3d_{z^2})^0$, was predicted to be favored by approximately 1.76 eV with respect to the singlet closed shell species, *i.e.* formally $(3d_{xy})^2$, $(3d_{xz})^2$, $(3d_{yz})^0$, $(3d_{x^2-y^2})^0$, $(3d_{z^2})^0$, in their respective equilibria. All ground state calculations were performed at the (unrestricted) density functional level of theory (DFT) by means of the PBE0 XC functional.[4] The def2-SVP[5,6] basis as well as the respective core potentials were applied for all atoms. A subsequent vibrational analysis was carried out for each optimized ground state structure (singlet and triplet) to verify that a minimum on the potential energy (hyper-)surface (PES) was obtained. Further equilibrium structures, such are more compact and bundled structures that might be involved potentially in excited state electron transfer processes from the BODIPY dyes to the manganese center of the POM were intentionally not investigated in this initial joint synthetic-spectroscopic-theoretical investigation.

Excited state properties such as excitation energies, oscillator strengths and electronic characters were calculated exclusively within the energetically favored triplet equilibrium structures at the time-dependent DFT (TDDFT) level of theory. Therefore, the 200 lowest spin-allowed triplet-triplet excited states were calculated. Thereby, the same XC functional, basis set and core potentials were applied as for the preceding ground state calculations. Noteworthy, TDDFT lacks accuracy for describing multiconfigurational systems and boron-species in general. Benchmark simulations evaluating the accuracy of these TDDFT results, as obtained by a manifold of functionals, with respect to state-of-the-art *ab initio* methods, *e.g.* multi-state restricted active space perturbation theory through second-order on a restricted active space self-consistent field reference (MS-RASPT2//RASSCF), have been performed, while the PBE0 functional was found to yield a satisfactory computational description of the low-lying excited states in BODIPY dyes.[7–12] Therefore, TD-PBE0 was applied in the present joint synthetic-spectroscopic-theoretical study as, unfortunately, high level *ab initio* methods are computationally to demanding in case of the present dyad. Effects of interaction with a solvent (acetonitrile: ε = 35.688, n = 1.344) were taken into account on the ground and excited states properties by the solute electron density (SMD) variant of the integral equation formalism of the polarizable

continuum model.[13,14] The non-equilibrium procedure of solvation was used for the calculation of the excitation energies within the Franck-Condon region of the respective redox species, which is well adapted for processes where only the fast reorganization of the electronic distribution of the solvent is important. All calculations were performed including D3 dispersion correction with Becke-Johnson damping.[15]

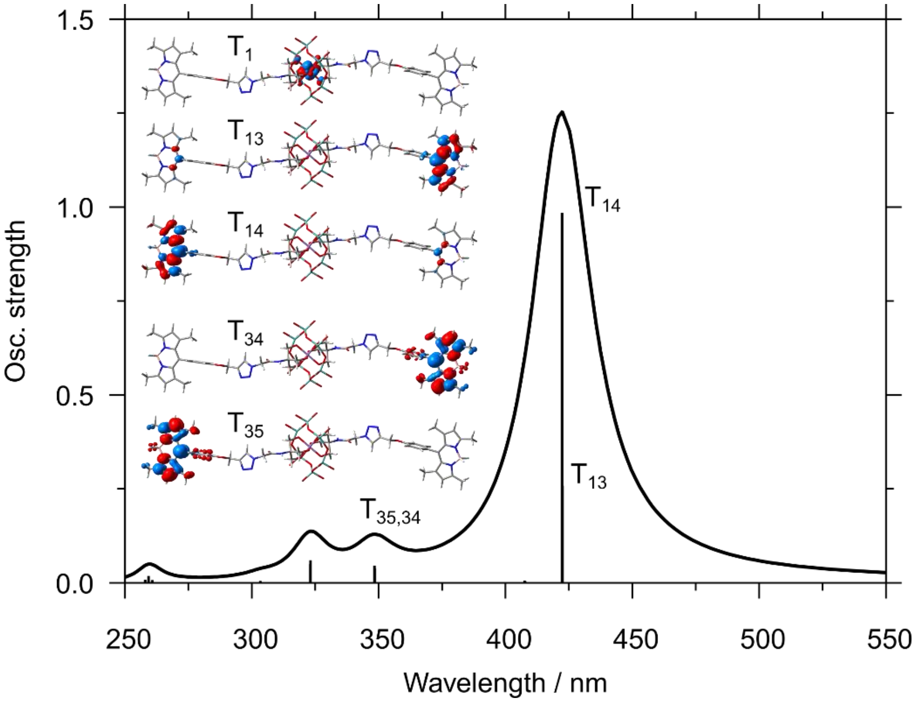

Figure S16. Simulated electronic absorption spectrum of BODIPY-POM **3** obtained at the TDDFT (unrestricted PBE0/def2-svp) level of theory in acetonitrile. States involved in the initial absorption are indicated. The electronic nature of the triplet ground state (T_1) and of spin and dipole-allowed triplet-triplet excitations of interest (into T_{13}, T_{14}, T_{34} and T_{35}) are illustrated by the spin density and charge density differences, respectively; charge transfer takes place from red two blue.

Table S3. Electronic properties, such as electronic characters based on the charge density differences (CDDs, charge transfer occurs from red to blue) excitation energies, excitation wavelengths, oscillator strengths, spin contamination and associated experimental wavelengths of the low-lying spin and dipole-allowed triplet-triplet excitations of BODIPY-POM **3** obtained at the TDDFT (unrestricted PBE0/def2-svp) level of theory in acetonitrile. States involved in the initial absorption and in the subsequent charge transfer are highlighted. The electronic nature of the triplet ground state (T_1) is illustrated by the spin density.

State	Charge density difference	E / eV	λ / nm	f	$\langle \hat{s}^2 \rangle$	λ_{exp} / nm
T_1 (^{3}MC)	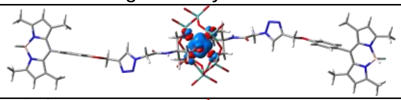	0.00	-	-	2.02	-
T_2 (^{3}MC)	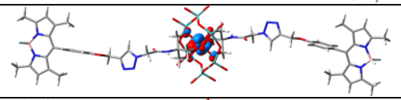	0.51	2437	0.0000	2.02	-
T_3 (^{3}MC)	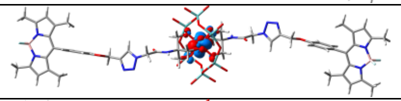	0.51	2434	0.0000	2.02	-
T_8 (^{3}MC)	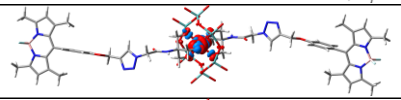	2.33	532	0.0000	2.02	-
T_9 (^{3}MC)	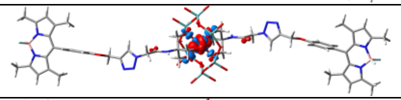	2.33	530	0.0000	2.02	-
T_{10} (^{3}MC)	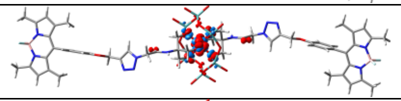	2.74	453	0.0000	2.03	-
T_{13} (^{3}IL)	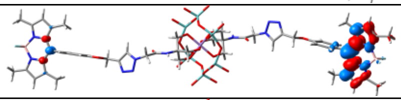	2.93	422	0.2584	2.02	500
T_{14} (^{3}IL)	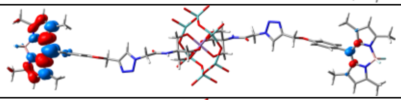	2.94	422	0.9851	2.02	500
T_{34} (^{3}IL)	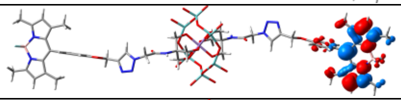	3.56	348	0.0385	2.02	-
T_{35} (^{3}IL)	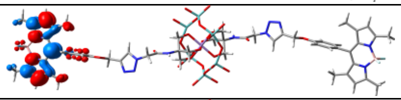	3.56	348	0.0461	2.02	-
T_{127} (^{3}LMCT)	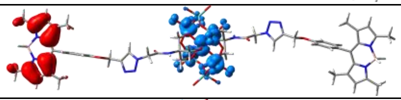	4.56	272	0.0000	2.65	-
T_{128} (^{3}LMCT)	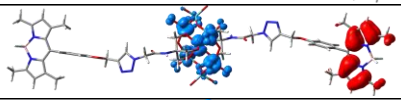	4.57	272	0.0000	2.65	-
T_{129} (^{3}LMCT)	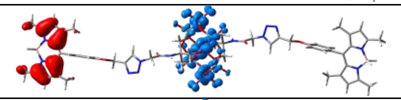	4.57	271	0.0000	2.61	-
T_{130} (^{3}LMCT)	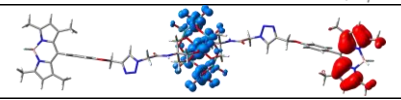	4.57	271	0.0000	2.61	

7. Literature references cited in SI

[1] S. Vanhaecht, J. Jacobs, L. Van Meervelt, T. N. Parac-Vogt, *Dalton Trans.* **2015**, *44*, 19059–19062.

[2] C. Zhang, J. Zhao, S. Wu, Z. Wang, W. Wu, J. Ma, S. Guo, L. Huang, *J. Am. Chem. Soc.* **2013**, *135*, 10566–10578.

[3] G. E. S. M. J. Frisch, G. W. Trucks, H. B. Schlegel, V. B. M. A. Robb, J. R. Cheeseman, G. Scalmani, A. V. M. G. A. Petersson, H. Nakatsuji, X. Li, M. Caricato, H. P. H. J. Bloino, B. G. Janesko, R. Gomperts, B. Mennucci, D. W.-Y. J. V. Ortiz, A. F. Izmaylov, J. L. Sonnenberg, A. P. F. Ding, F. Lipparini, F. Egidi, J. Goings, B. Peng, N. R. T. Henderson, D. Ranasinghe, V. G. Zakrzewski, J. Gao, R. F. G. Zheng, W. Liang, M. Hada, M. Ehara, K. Toyota, H. N. J. Hasegawa, M. Ishida, T. Nakajima, Y. Honda, O. Kitao, J. E. P. T. Vreven, K. Throssell, J. A. Montgomery, Jr., et al., *Gaussian 16, Revis. A.03, Gaussian, Inc., Wallingford CT* **2016**.

[4] C. Adamo, V. Barone, *J. Chem. Phys.* **1999**, *110*, 6158–6170.

[5] F. Weigend, R. Ahlrichs, *Phys. Chem. Chem. Phys.* **2005**, *7*, 3297.

[6] F. Weigend, *Phys. Chem. Chem. Phys.* **2006**, *8*, 1057.

[7] M. R. Momeni, A. Brown, *J. Chem. Theory Comput.* **2015**, *11*, 2619–2632.

[8] J. D. Spiegel, M. Kleinschmidt, A. Larbig, J. Tatchen, C. M. Marian, *J. Chem. Theory Comput.* **2015**, *11*, 4316–4327.

[9] S. Chibani, A. D. Laurent, B. Le Guennic, D. Jacquemin, *J. Chem. Theory Comput.* **2014**, *10*, 4574–4582.

[10] S. Ji, J. Ge, D. Escudero, Z. Wang, J. Zhao, D. Jacquemin, *J. Org. Chem.* **2015**, *80*, 5958–5963.

[11] L. Dura, M. Wächtler, S. Kupfer, J. Kübel, J. Ahrens, S. Höfler, M. Bröring, B. Dietzek, T. Beweries, *Inorganics* **2017**, *5*, 21.

[12] K. Ziems, S. Gräfe, S. Kupfer, *Catalysts* **2018**, *8*, 520.

[13] A. V. Marenich, C. J. Cramer, D. G. Truhlar, *J. Phys. Chem. B* **2009**, *113*, 6378–6396.

[14] A. Iagatti, L. Cupellini, G. Biagiotti, S. Caprasecca, S. Fedeli, A. Lapini, E. Ussano, S. Cicchi, P. Foggi, M. Marcaccio, et al., *J. Phys. Chem. C* **2016**, *120*, 16526–16536.

[15] S. Grimme, S. Ehrlich, L. Goerigk, *J. Comput. Chem.* **2011**, *32*, 1456–1465.
